# Supplementary material for: Trends in extent of surgical cytoreduction for patients with ovarian cancer
Source: PLoS One. 2021 Dec 8;16(12):e0260255. doi: 10.1371/journal.pone.0260255 (PMC8654234; doi:10.1371/journal.pone.0260255)
Supplement: S1 Table — (DOCX) [file pone.0260255.s001.docx]

**S1 Table: International Classification of Diseases (ICD) codes used for patient identification and characterization.**

|  | **ICD-9 Codes** | **ICD-10 codes** |
| --- | --- | --- |
| Ovarian cancer | 183.0, 183.2, 158.8, 158.9 | C481, C482, C488, C561, C562, C569, C5700, C5701, C5702 |
| Procedure involving oophorectomy | 65.31, 65.39, 65.41, 65.49, 65.51, 65.52, 65.53, 65.54, 65.61, 65.62, 65.63, 65.64, 68.8 | 0UT00ZZ, 0UT04ZZ, 0UT07ZZ, 0UT08ZZ, 0UT0FZZ, 0UT10ZZ, 0UT14ZZ, 0UT17ZZ, 0UT18ZZ, 0UT1FZZ, 0UT20ZZ, 0UT24ZZ, 0UT27ZZ, 0UT28ZZ, 0UT2FZZ |
| Small bowel resection | 45.61, 45.62, 45.63, 45.91 | 0DB80ZZ, 0DB83ZZ, 0DB84ZZ, 0DB87ZZ, 0DB88ZZ, 0DT90ZZ, 0DT94ZZ, 0DT97ZZ, 0DT98ZZ, 0DTA0ZZ, 0DTA4ZZ, 0DTA7ZZ, 0DTA8ZZ, 0DTB0ZZ, 0DTB4ZZ, 0DTB7ZZ, 0DTB8ZZ, 0DT80ZZ, 0DT84ZZ, 0DT87ZZ, 0DT88ZZ, 0D190Z9, 0D190ZA, 0D190ZB, 0D194Z9, 0D194ZA, 0D194ZB, 0D198Z9, 0D198ZA, 0D198ZB, 0D1A0ZA, 0D1A0ZB, 0D1A4ZA, 0D1A4ZB, 0D1A8ZA, 0D1A8ZB, 0D1A8ZH, 0D1B0ZB, 0D1B4ZB, 0D1B8ZB, 0D1B8ZH |
| Colon resection | 45.71, 45.72, 45.73, 45.74, 45.75, 45.76, 45.79, 45.81, 45.82, 45.83, 45.93, 45.94, 17.31, 17.32, 17.33, 17.34, 17.35, 17.36, 17.39 | 0DBE0ZZ, 0DBE3ZZ, 0DBE7ZZ, 0DBE8ZZ, 0DTH0ZZ, 0DTH7ZZ, 0DTH8ZZ, 0DTF0ZZ, 0DTF7ZZ, 0DTF8ZZ, 0DTK0ZZ, 0DTL0ZZ, 0DTL7ZZ, 0DTL8ZZ, 0DTLFZZ, 0DTG0ZZ, 0DTG7ZZ, 0DTG8ZZ, 0DTGFZZ, 0DTN0ZZ, 0DTN7ZZ, 0DTN8ZZ, 0DTNFZZ, 0DBGFZZ, 0DBLFZZ, 0DBMFZZ, 0DBNFZZ, 0DTMFZZ, 0DBE4ZZ, 0DTH4ZZ, 0DTF4ZZ, 0DTL4ZZ, 0DTG4ZZ, 0DTN4ZZ, 0DBE4ZZ |
| Rectosigmoid resection | 45.76, 48.50, 48.51, 48.52, 48.59, 48.61, 48.62, 48.63, 48.64, 48.65, 48.69 | 0DTN0ZZ, 0DTN7ZZ, 0DTN8ZZ, 0DTNFZZ, 0D1N0Z4, 0DTP0ZZ, 0DTP4ZZ, 0DTP7ZZ, 0DTP8ZZ, 0DTN4ZZ, 0D1N4Z4, 0DBP0ZZ, 0DBP4ZZ |
| Liver resection | 50.22, 50.3, 50.4 | 0FB00ZZ, 0FB03ZZ, 0FB04ZZ, 0FT10ZZ, 0FT14ZZ, 0FT20ZZ, 0FT24ZZ, 0FT00ZZ, 0FT04ZZ |
| Bladder resection | 57.6, 57.71, 57.79 | 0TBB0ZZ, 0TBB3ZZ, 0TBB4ZZ, 0TTB0ZZ, 0TTB4ZZ, 0TTB7ZZ, 0TTB8ZZ |
| Diaphragm resection | 34.81, 34.84 | 0BBT0ZZ, 0BBT3ZZ, 0BBT4ZZ, 0BQT0ZZ, 0BQT3ZZ, 0BQT4ZZ |
| Spleen resection | 41.43, 41.5 | 07BP0ZZ, 07BP3ZZ, 07BP4ZZ, 07TP0ZZ, 07TP4ZZ |
| Gastric resection | 43.41, 43.42, 43.49, 43.5, 43.6, 43.7, 43.81, 43.82, 43.89, 43.91, 43.99 | 0D564ZZ, 0D568ZZ, 0DB64ZZ, 0DB68ZZ, 0DB60ZZ, 0DB63ZZ, 0DB67ZZ, 0D560ZZ, 0D563ZZ, 0D567ZZ, 0DB40ZZ, 0DB43ZZ, 0DB44ZZ, 0DB47ZZ, 0DT40ZZ, 0DT44ZZ, 0DT47ZZ, 0DT48ZZ, 0DT70ZZ, 0DT74ZZ, 0DT77ZZ, 0DT78ZZ, 0D168ZA, 0D160ZA, 0D164ZA, 0DB64Z3 |
| Ileostomy | 46.01, 46.20, 46.21, 46.22, 46.23, 46.24 | 0D190Z4, 0D194Z4, 0D198Z4, 0D1A0Z4, 0D1A4Z4, 0D1A8Z4, 0D1B8Z4, 0D1B0Z4, 0D1B4Z4, 0D1B8Z4 |
| Colostomy | 46.10, 46.11, 46.13, 46.14 | 0D1H0Z4, 0D1H4Z4, 0D1H8Z4, 0D1K0Z4, 0D1K4Z4, 0D1K8Z4, 0D1L0Z4, 0D1L4Z4, 0D1L8Z4, 0D1N0Z4, 0D1N4Z4, 0D1N8Z4 |
