# Supplementary material for: Trends in extent of surgical cytoreduction for patients with ovarian cancer
Source: PLoS One. 2021 Dec 8;16(12):e0260255. doi: 10.1371/journal.pone.0260255 (PMC8654234; doi:10.1371/journal.pone.0260255)
Supplement: Attachment — Submitted filename: Response to Reviewers.doc [file pone.0260255.s002.doc]

**David Geffen School of Medicine at UCLA**

**Ronald Reagan-UCLA Medical Center**

Department of Obstetrics and Gynecology

Division of Gynecologic Oncology

200 Medical Plaza, Suite 220

Los Angeles, CA 90095

Phone: 310-794-9098

Fax: 310-794-7110

JGCohen@mednet.ucla.edu

Joshua G. Cohen, MD, FACOG, FACS

Assistant Professor, Division of Gynecologic Oncology

Department of Obstetrics and Gynecology

David Geffen School of Medicine at UCLA

June 10, 2021

Helge Bruns, MD

Editor-in-Chief
PLOS ONE

**RE: Revisions for Manuscript entitled, “Trends in Extent of Surgical Cytoreduction for Patients with Ovarian Cancer”**

Dear Dr. Bruns:

On behalf of my co-authors, we are appreciative of the detailed and thoughtful reviews rendered on our manuscript. We have addressed each reviewer’s comments below:

**Reviewer #1:**

**Data from NIS has been analyzed in order to examine the trend of ECR for patients with ovarian cancer. The reviewer has some minor points that need to be addressed:**

**1. Has there been exclusion criteria? Was invalid, duplicated or missing data detected?**

In our initial analysis, we excluded all patients who were less that 18 years of age but did not exclude patients based on the availability of mortality or demographic information, as these were not directly related to our conclusions regarding the utilization of extended cytoreduction. We have since performed a post-hoc analysis of our study population to exclude patients missing these variables. We identified 6 unweighted hospitalizations that were missing age or mortality information, and the exclusion of these patients did not change any of our significant findings or conclusions.

**2. In 2015 a transition to ICD 10 took place due to update of a coding system. Has this database change affected the data collected for this research? Has the algorithm for data collection been modified?**

We carefully adjusted our analysis to account for the transition to ICD-10 coding, which took place in October of 2015. We included both ICD-9 and -10 codes for our data collection and definition of diagnoses and procedures. To test whether this transition would affect our trend analysis, we performed chi square analysis to compare the proportion of procedures occurring in the third and fourth quarters of 2015. We performed this analysis for the proportion of our population receiving extended cytoreduction as well as those receiving each of the four procedures that were analyzed separately (small bowel resection, colon resection, rectosigmoid resection, and colostomy formation) and found no significant differences between the data for the third and fourth quarters of 2015. We have added a description of this analysis to our methods section (see page 6, lines 83-89).

**3. Although patient characteristics are considered in the study objective, authors have not included this aspect in their hypothesis.**

**The authors hypothesize, that ECR is associated with a higher risk of perioperative mortality and length of stay. It is very likely that this effect will occur, when comparing extended surgery procedures against minor ones.**

We thank the reviewer for identifying this area for improvement, and we have added more detail to our hypothesis. We hypothesized that additional factors, including patient income and disease burden, would also contribute to their access to extended cytoreduction (see page 5, lines 60-63).

**4. Please describe how the reference groups in the multivariate regression were determined.**

The reference groups were initially chosen based on clinical relevance and ability to test our hypotheses. However, we appreciate the opportunity to employ a more systematic approach to reference group selection. We have adjusted our analysis so that the reference group in multivariable regression is the majority group within each category (TABLE 2), have added a sentence to describe this decision in our methods (see page 7, lines 117-118) and have changed the wording and findings of our abstract and results accordingly.

**5. In order to determine if the relative number of ECR procedures has decreased 2013- 2017 it is necessary to indicate the number of new diagnosed ovarian cancer per year. The decrease of the absolute ECR number is most significant since 2016. This coincides with the database change mentioned in item 2 above. Could this have interfered in the results?**

Unfortunately, the NIS provides only inpatient data and does not allow us to track patients across multiple hospitalizations, so we are unable to determine the number of newly diagnosed ovarian cancer cases per year. According to publicly available SEER data, the rate of ovarian cancer diagnosis has decreased in recent years, and this may contribute to an absolute decrease in the number of patients undergoing any surgery for ovarian cancer. We have added this limitation to our discussion section (see page 14, lines 237-240). However, our analysis focuses not on the decrease in the absolute number of surgeries performed, but rather on the decrease in the proportion of these surgeries that involved extended cytoreduction, which should be unaffected by national trends in ovarian cancer incidence. We have adjusted our trend analysis from the nonparametric test to the Cochrane-Armitage test for trend in proportions (Figures 1, 2, and 3) so that the absolute number of procedures should not affect our finding. Regarding the 2015 coding change from ICD-9 to ICD-10, we adjusted our analysis and checked for differences as described in answer #2. We are confident based on our finding of no change between Q3 and Q4 of 2015 that this database change did not significantly affect our trend analysis and have added a brief description to our methods section (see page 6, lines 83-89).

**6. Which data in this research supports the statement that socioeconomic factors influence the access of surgery? Or is this statement supported by a citation?**

In this research, we used the income quartile of the patient’s ZIP Code to as a proxy for socioeconomic factors and found that those of higher income quartiles were more likely to have ECR. Other studies have made conclusions that income affects whether patients undergo ovarian cancer surgery (1, 2). However, we agree with the reviewer that while the highest income quartile do have a higher rate of ECR surgery, there are other factors that contribute to the rate of ECR.

1. Goldberg, E.M., Berger, Y., Sood, D. et al. Differences in Sociodemographic Disparities Between Patients Undergoing Surgery for Advanced Colorectal or Ovarian Cancer. Ann Surg Oncol (2021). https://doi.org/10.1245/s10434-021-10086-y

2. Long B, Chang J, Ziogas A, Tewari KS, Anton-Culver H, Bristow RE. Impact of race, socioeconomic status, and the health care system on the treatment of advanced-stage ovarian cancer in California. Am J Obstet Gynecol. 2015 Apr;212(4):468.e1-9. doi: 10.1016/j.ajog.2014.10.1104. Epub 2014 Oct 31. PMID: 25448522.

**7. The increase of neoadjuvant chemotherapy has been observed (cited) between 2004 and 2013. Does recent data support this trend? Moreover the observation in the reviewed research starts at 2013.**

As suggested by the reviewer, there continues to be an increase in neoadjuvant chemotherapy in more recent data, even beyond 2013. We have cited more recent studies within our manuscript to reflect this (see page 14, lines 213-214). A limitation of this database is being unable to identify patients who had underwent neoadjuvant chemotherapy. Given the decrease in ECR over time, we agree this may be due to the increased use of neoadjuvant chemotherapy and warrants further study.

**8. The decrease in bowel resection could reflect the increasing use of neoadjuvant therapy, but there are also other explanations. As previously mentioned the number of new diagnosed ovarian cancer is needed in order to confirm a decrease in bowel resection.**

We agree that knowledge of the absolute number of newly diagnosed ovarian cancer cases would be an interesting and helpful addition to our analysis. However, as previously described, we are unfortunately unable to determine this number using the NIS database. Nonetheless, we believe it to be a significant and clinically relevant finding that, among patients undergoing ovarian cancer surgery, the proportion having bowel resection or several other components of extended cytoreduction has decreased. We have adjusted the wording of our analysis to include the potential for other explanations for this trend (see page 14, lines 237-240) and have added our inability to determine the number of new cases per year to our limitations (see page 16, lines 268-270).

**9. How the decrease of ECR relates to the quality of care provided to ovarian cancer patient?**

Quality metrics in surgery include rates of optimal cytoreduction, for which to achieve optimal cytoreduction, these ECR surgeries are not uncommonly required. We agree with the reviewer that future areas of study are needed to analyze these quality metrics to assess whether decreased ECR is associated with worse overall survival based on overall tumor debulking.

**Reviewer #2: I found this manuscript excellent and very interesting to read. It highlights differences in the management of ovarian cancer depending on certain demographic and hospital characteristics.**

**The only correlation that is difficult to understand is how patients with increased comorbidities had more chances of undergoing extensive surgery for ovarian cancer. The index used in this study is complex with a lot of parameters contributing to the final score and this might account for the finding. If the PS was available it would have made more sense in interpreting the comorbidity index used in this study.**

**Also I am not familiar with the data base used for extracting data for this study, however its limitations are thoroughly discussed by the author and I don't think these are reducing the value of the results.**

**I think it should be published at its current form in PLOS ONE**

We thank the reviewer for their kind remarks regarding our study. We understand the confusion regarding comorbidities and their correlation to ECR. This is likely attributable to the specific comorbidities that constitute the Elixhauser Comorbidity Index, including weight loss and metastatic disease, which may be directly related to tumor burden and thus reflective of the need for more extensive tumor debulking.

We sincerely appreciate the comments made by the reviewers and hope that we have satisfactorily addressed them. We have included a copy of our manuscript with highlighted changes. We believe these comments allowed for the creation of an improved presentation of our study. Please feel free to contact me for any further questions or comments.

Most sincerely yours,

Joshua G. Cohen, MD, FACOG, FACS

Assistant Professor, Division of Gynecologic Oncology

Department of Obstetrics and Gynecology

David Geffen School of Medicine at UCLA
